# Differential Postoperative Effects of Volatile Anesthesia and Intraoperative Remifentanil Infusion in 7511 Thyroidectomy Patients: A Propensity Score Matching Analysis

**DOI:** 10.1097/MD.0000000000002764

**Published:** 2016-02-18

**Authors:** Jun-Young Jo, Seong-Soo Choi, Jung Min Yi, Eun Young Joo, Ji Hyun Kim, Se Ung Park, Ji-Hoon Sim, Myong-Hwan Karm, Seungwoo Ku

**Affiliations:** From the Department of Anesthesiology and Pain Medicine, Asan Medical Center, University of Ulsan College of Medicine, Seoul, Korea.

## Abstract

Although remifentanil is used widely by many clinicians during general anesthesia, there are recent evidences of opioid-induced hyperalgesia as an adverse effect. This study aimed to determine if intraoperative remifentanil infusion caused increased pain during the postoperative period in patients who underwent a thyroidectomy.

A total of 7511 patients aged ≥ 20 years, who underwent thyroidectomy between January 2009 and December 2013 at the Asan Medical Center were retrospectively analyzed. Enrolled patients were divided into 2 groups: group N (no intraoperative remifentanil and only volatile maintenance anesthesia) and group R (intraoperative remifentanil infusion including total intravenous anesthesia and balanced anesthesia). Following propensity score matching analysis, 2582 patients were included in each group. Pain scores based on numeric rating scales (NRS) were compared between the 2 groups at the postoperative anesthetic care unit and at the ward until 3 days postoperation. Incidences of postoperative complications, such as nausea, itching, and shivering were also compared.

The estimated NRS pain score on the day of surgery was 5.08 (95% confidence interval [CI] 4.97–5.19) in group N patients and 6.73 (95% CI 6.65–6.80) in group R patients (*P* < 0.001). There were no statistically significant differences in NRS scores on postoperative days 1, 2, and 3 between the 2 groups. Postoperative nausea was less frequent in group R (31.4%) than in group N (53.5%) (*P* < 0.001). However, the incidence of itching was higher in group R (4.3%) than in group N (0.7%) (*P* < 0.001).

Continuous infusion of remifentanil during general anesthesia can cause higher intensity of postoperative pain and more frequent itching than general anesthesia without remifentanil infusion immediately after thyroidectomy. Considering the advantages and disadvantages of continuous remifentanil infusion, volatile anesthesia without opioid may be a good choice for minor surgeries, such as thyroidectomy.

## INTRODUCTION

Nontrivial anesthesiologists refuse volatile induction and maintenance of anesthesia (VIMA) due to the development of high-quality intravenous anesthetics such as propofol and remifentanil. Remifentanil, a potent, fast acting synthetic opioid and a direct μ-opioid receptor agonist,^1^ is typically used as a high-quality anesthesia for major surgeries. The pharmacokinetic characteristics of remifentanil (short onset time, terminal elimination time, and context-sensitive half-time) are not affected by liver and kidney function.^1–4^ Therefore, remifentanil can be used in various types of patients without remnant opioid adverse effects such as respiratory depression and delayed awakening after surgery.^4,5^ However, opioid-induced hyperalgesia (OIH) is considered to be a problem with remifentanil infusion during postoperative pain management.^5–9^

OIH has been well established in animal studies,^10–12^ but, there are still discrepancies among the results of human clinical studies.^13–19^ Some reports have suggested that remifentanil causes acute opioid tolerance and hyperalgesia.^13,19^ However, these studies involved patient-controlled analgesics with morphine for postoperative pain management. Other clinical studies have reported no clinical evidence of acute opioid tolerance after remifentanil infusion.^15,16,18^ However, these studies used relatively low doses remifentanil, making it difficult to determine the existence of OIH.^15,16^

The objective of our present study was to investigate pain intensity and the needs for rescue analgesics during the postoperative period in patients with or without a continuous infusion of remifentanil during a thyroidectomy, which is a relatively minor surgery. Secondary outcome measures included the incidence of postanesthetic complications such as postoperative nausea or vomiting, and itching.

## METHODS

### Study Population

We obtained approval from the Institutional Review Board of Asan Medical Center, Seoul, Korea (2014–1066) and retrospectively assessed 7511 American Society of Anesthesiologists physical status I–II patients who underwent a thyroidectomy without neck dissection between January 2009 and December 2013 at our institution. Patients under 20 years of age or with known allergies to opioids, airway problems, or kidney diseases were excluded. Exclusion criteria also included opioid use in the month prior to surgery or chronic pain syndrome. Data from patients who experienced reoperations or intensive care due to postoperative complications were also excluded.

### Anesthetic Management and Design

Enrolled patients were divided into 2 groups, with (group R) and without (group N) intraoperative remifentanil infusion. The attending anesthesiologists decided whether to administer remifentanil or not. In group N, induction of anesthesia was performed with inhalant anesthetics, such as sevoflurane, or intravenous anesthetics, such as pentothal sodium (5 mg/kg) or propofol (2 mg/kg). Rocuronium (0.6 mg/kg) or vecuronium (1.2 mg/kg) was administered for tracheal intubation. In group R, anesthesia was induced with pentothal sodium (5 mg/kg) or propofol (2 mg/kg). After loss of consciousness, rocuronium (0.6 mg/kg) or vecuronium (1.2 mg/kg) was administered and tracheal intubation was performed. In group N, anesthesia was maintained with volatile anesthetics (nitrous oxide and 50% oxygen). In group R, remifentanil was infused continuously with inhalant anesthetics or a continuous infusion of propofol. Remifentanil was continuously administered via a target-controlled infusion (TCI) pump. The remifentanil dose was adjusted to maintain the mean blood pressure to within 20% of the baseline. Patients undergoing total intravenous anesthesia were administered 50 to 100 μg fentanyl ∼30 minutes before the end of surgery to prevent extreme pain at the end of remifentanil injection when the surgery was over. At the end of the operation, inhalant anesthetics or infused propofol and remifentanil were discontinued. Respiratory movement was allowed to recover spontaneously, and 15 mg pyridostigmine and 0.4 mg glycopyrrolate were administered intravenously to reverse muscle relaxation. Endotracheal tubes were removed when patients regained consciousness with spontaneous respiration. Patients were then transferred to the postanesthetic care unit and scored for pain. If the pain score is >5 at the postanesthetic care unit, the anesthesiologists usually order rescue analgesics, either opioid or nonsteroidal anti-inflammatory drugs (NSAIDs), according to their preferences. Pain scores were based on numeric rating scales (NRS) for pain at the postoperative anesthetic care unit and at the ward until postoperative day 3. An 11-point numerical rating scale (NRS; 0 = no pain, 10 = unbearable pain) was used to assess postoperative pain intensity at the postoperative anesthetic care unit and at the ward until postoperative day 3. The means of the highest NRS of scores for each day were compared between the 2 groups along with incidences of postoperative complications. Demographic data and information about anesthetic management, pain intensity, need for rescue analgesics, and various complications were collected from electronic hospital databases.

### Statistical Analysis

Before propensity score matching, continuous variables are expressed as the mean ± standard deviation and compared using the *t* test. Categorical variables are expressed as frequencies with percentages, and compared using the χ^2^ test or Fisher exact test, as appropriate. A 1:1 propensity score matching was used to overcome individual and surgical differences between the 2 groups. To account for potential confounding due to systematic differences between the 2 groups and to minimize the selection bias on outcome, we matched each patient on baseline age, gender, height, weight, and type of operation. After matching, demographic data were compared using the Wilcoxon signed rank test for continuous variables and the McNemar test for categorical variables. As data loss due to missing value from electronic database was expected, the linear mixed effect model (LMEM) was used to compare changes within and between the groups in terms of the NRS scores on postoperative days 0, 1, 2, and 3. Compared with analysis of variance, the LMEM is more flexible in accommodating longitudinal data features and is more efficient in compensating for missing data. Complication incidence was analyzed by the McNemar test. All data manipulations and statistical analyses were performed using SPSS for Windows, version 21 (IBM corp, Armonk, NY) and Stata software version 13.1 (StataCorp LP, College Station, TX). All reported *P* values are 2-sided, and *P* values < 0.05 were considered statistically significant.

## RESULTS

**FIGURE 1:**
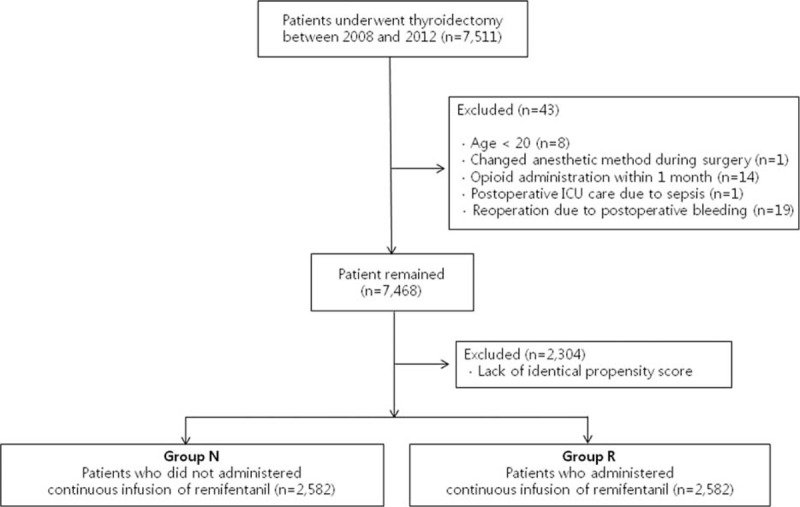
Flow diagram of the inclusion criteria, exclusion criteria, and study design.

Between January 2009 and December 2013, a total of 7511 patients with American Society of Anesthesiologists physical status I, II, and III underwent thyroidectomy without neck dissection at the Asan Medical Center. Eight patients were <20 years old and 14 patients were administered opioids in the month prior to the operation. Nineteen patients required a reoperation due to bleeding. Two patients were excluded; one was changed to the anesthetic method of treatment, and the other needed postoperative intensive care due to sepsis. Finally, raw data from 7468 patients were analyzed (Figure 1).

The baseline and perioperative characteristics of this study population are presented in Table 1. There were 3372 patients in group N (no remifentanil infusion) and 4096 patients in group R (remifentanil infusion). There were statistically significant differences in height, American Society of Anesthesiologists physical status, type of operation, and type of anesthetic agent between the 2 groups. Patients were matched and propensity score were estimated via multivariable logistic regression model. After matching, there were a total of 2582 patients in each group (Figure 1), with no differences between the 2 groups in terms of demographic data with the exception of anesthetic agents (Table 2).

**TABLE 1:**
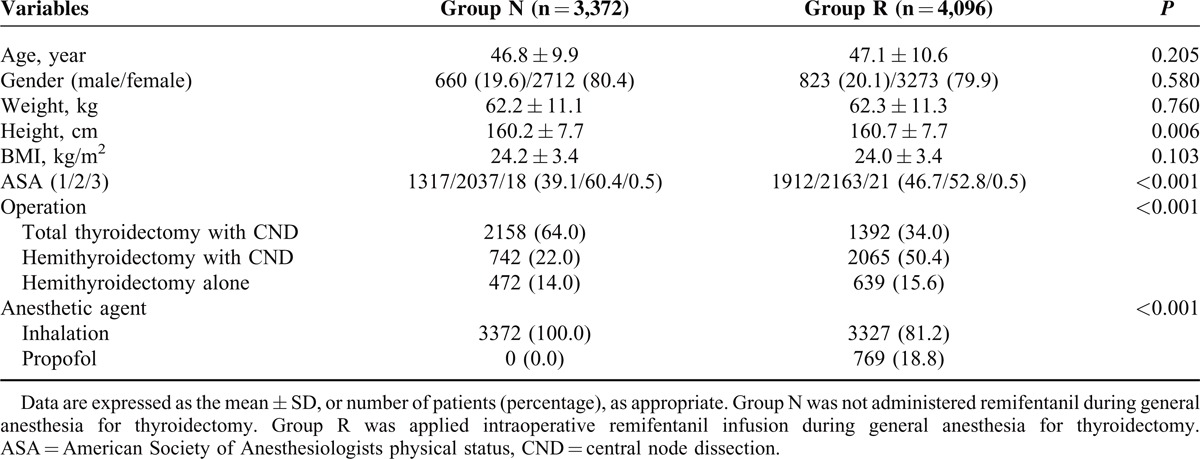
Characteristics of Study Population

**TABLE 2:**
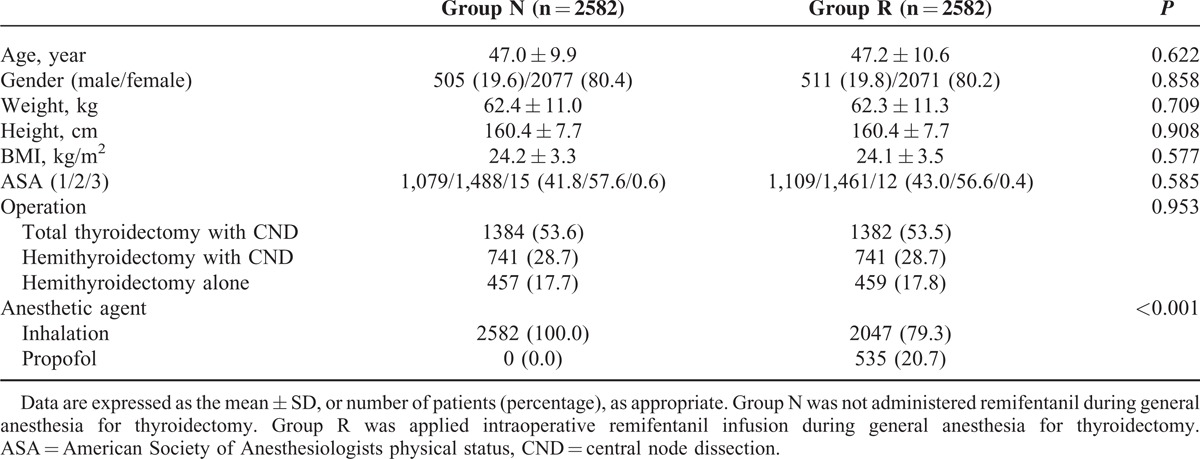
Characteristics of Study Population After the Propensity Score Matching

Postoperative pain intensity values are listed in Table 3. The NRS pain scores were compared between the 2 groups using the LMEM with time as the random effect and group as the fixed effect. The estimated mean NRS on postoperative day 0, which was estimated at the postanesthetic care unit, was 5.08 (95% confidence interval [CI] 4.97–5.19] in group N patients and 6.73 (95% CI 6.65–6.80) in group R patients. The estimated difference in the mean values on the day of surgery between the 2 groups was 1.64 (95% CI 1.51–1.77; *P*value < 0.001). The Mean NRS score for group N were 2.70 (95% CI 2.63–2.76), 1.65 (95% CI 1.57–1.72), and 0.83 (95% CI 0.74–0.91) on postoperative days 1, 2, and 3, respectively. NRS scores for group R were 2.53 (95% CI 2.46–2.60), 1.64 (95% CI 1.55–1.73), and 0.87 (95% CI 0.73–1.00) on postoperative days 1, 2, and 3 in patients of group R, respectively. The estimated difference in the mean values on postoperative day 1 was −0.16 (95% CI −0.25 to 0.07; *P* value = 0.001). No differences were observed on postoperative days 2 and 3. Additionally, more patients in group R administered rescue analgesics at the postanesthetic care unit than patients in group N (84.8% vs. 32.6%; *P* value < 0.001). On the other hand, the rescue analgesics administered at ward varied depending on the preference of surgeons among NSAIDs, acetaminophen, and tramadol but not opioids. However, there was no statistical difference between the ratios of need for rescue analgesics in 2 groups at ward.

**TABLE 3:**
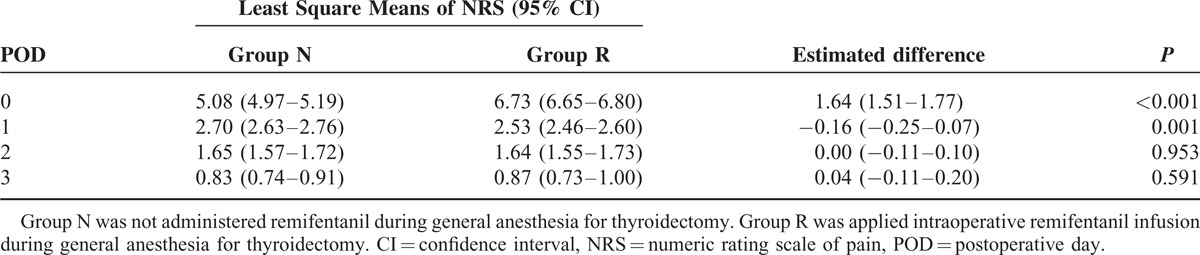
Least Square Means of Numeric Rating Scale of Pain and Differences Between 2 Groups

Incidences of each postoperative complication are presented in Table 4. Patients in both groups complained of nausea, shivering, urticaria, and itching and minor bleeding that did not require reoperation or desaturation. Significantly more patients in group N complained of nausea compared with group R (53.3% vs 31.4%; *P* < 0.001). However significantly more patients in group R complained of urticaria or itching, compared with group N (4.4% vs 0.7%; *P* < 0.001).

**TABLE 4:**
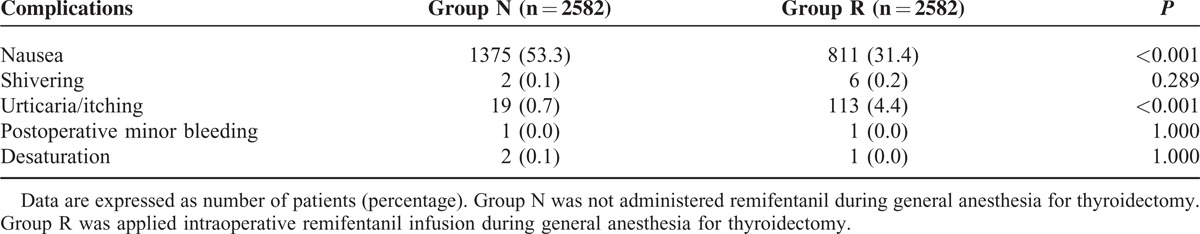
Comparison of the Incidence of Complications

## DISCUSSION

In the present study, we found that intraoperative remifentanil infusion increased the postoperative pain intensity on the day of surgery which required rescue analgesics. On postoperative day 1, the pain intensity in group N was slightly higher than that in group R, but this had no clinical implication. The increased pain intensity in group R on the day of surgery can be explained by OIH due to remifentanil-based anesthesia. OIH and acute opioid tolerance have been well established through multiple animal studies,^7,10–12^ although their relevance to humans and the exact underlying mechanisms are still unclear.^6–8,13,15–19^ Several studies have suggested that remifentanil causes OIH. For example, in patients undergoing major abdominal surgery, Guignard et al have demonstrated that intraoperative administration of relatively large remifentanil doses (0.3 μg/kg/min of mean) increased postoperative pain and morphine consumption in the first postoperative day.^13^ In addition, Hansen et al showed that a high dose of remifentanil (0.4 μg/kg/min) added to combined general and epidural anaesthesia could induce OIH and/or acute opioid tolerance after major abdominal surgery at the immediate postoperative period (0–2 h).^19^ Our current findings for OIH after intraoperative remifentanil infusion are consistent with these earlier studies. On the other hand, several reports have found no clinical evidence of acute opioid tolerance after intraoperative infusion of remifentanil. Cortinez et al revealed that intraoperative continuous remifentanil infusion (0.25 μg/kg/min) during gynecological surgery did not increase postoperative morphine consumption during the first 24 hours, compared to sevoflurane based anesthesia without remifentanil infusion.^15^ Moreover, Lee et al reported that there were no differences in postoperative opioid consumptions and pain intensities during the first 24 h in patients undergoing isoflurane anesthesia with 70% nitrous oxide or with 0.17 μg/kg/min remifentanil infusion for open colorectal surgery.^18^ However, those studies used relatively low doses of remifentanil, which may have limited its influence on OIH.

According to previous reports, OIH and acute opioid tolerance cannot be discriminated clinically, although they may share a common mechanism involving the NMDA receptor.^7,8,10,20,21^ NMDA receptor antagonists such as ketamine or magnesium have been suggested for postoperative pain management.^22,23^ Strictly speaking, however, OIH and opioid tolerance are conceptually different. Opioid tolerance is a progressive decline in response to opioids, which increases demand. OIH is a paradoxical pain sensitization of the nervous system.^5,8^ Despite these differences, these conditions both result in high opioid demand and pain intensity.^8^ The retrospective nature of our present study did not allow us to specify the form of postoperative pain control and compare opioid demand. However, the need for rescue analgesics led us to speculate that OIH was playing a role. A prospective randomized study is needed to clarify this point.

Neither of our study groups was superior in terms of complications. Postoperative nausea and vomiting (PONV) was lower in group R, whereas itching and urticaria were lower in group N. Opioids and inhalant anesthetics are both known to be major risk factors for PONV,^24,25^ whereas propofol is known to reduce the degree of PONV.^26–29^ Approximately 20% of our group R patients received propofol for maintenance of anesthesia, which might explain the lower incidence of PONV in this group. In both groups, PONV and itching only occurred for a short period on the day of surgery or on postoperative day 1, suggesting minimal clinical implications.

Continuous administration of remifentanil during major operations has distinct advantages, such as maintenance of stable vital signs, applicability to patients with kidney and liver conditions, and reduced respiratory depression.^30^ However, in cases of minor surgery such as thyroidectomy, continuous infusion of remifentanil can be inconvenient and expensive. Therefore, volatile maintenance anesthesia without remifentanil infusion may represent a good choice for minor operations.

There were several limitations to our present study of note. First, there was no standardized postoperative pain management at the postanesthetic care unit or ward. Two surgical departments (otorhinolaryngology and general surgery) and 11 surgeons performed thyroidectomy in the present study, and each of these clinicians used their preferred method of pain management. Possible differences in surgical procedures also might have influenced the postoperative pain intensity scores. Second, we could not control for the dose of remifentanil. Several studies have suggested that only high doses of remifentanil infusion cause rapid opioid tolerance and/or hyperalgesia.^5,19,31^ Finally, due to the retrospective nature of our current analyses, we could not control the use of pre-emptive antiemetics by some of the surgeons, which might have affected the PONV incidence. A randomized controlled study is necessary in the future to overcome these limitations.

In conclusion, intraoperative remifentanil infusion during anesthesia can cause higher intensity of pain during the immediate postoperative period following thyroidectomy. Continuous infusion of remifentanil during general anesthesia in minor surgery may not be superior to volatile anesthesia without opioid in terms of overall complications. Volatile maintenance anesthesia without remifentanil is worth considering for minor operations.
